# Widely Targeted HPLC-MS/MS Metabolomics Analysis Reveals Natural Metabolic Insights in Insects

**DOI:** 10.3390/metabo13060735

**Published:** 2023-06-08

**Authors:** Zhaoxin Li, Yunlong Cheng, Jinxin Chen, Weijun Xu, Wentao Ma, Sheng Li, Erxia Du

**Affiliations:** 1 Guangdong Provincial Key Laboratory of Insect Developmental Biology and Applied Technology, Institute of Insect Science and Technology & School of Life Sciences, South China Normal University, Guangzhou 510631, China; 2020010172@m.scnu.edu.cn (Z.L.); 2021023014@m.scnu.edu.cn (Y.C.); 2021023016@m.scnu.edu.cn (J.C.); 2022023005@m.scnu.edu.cn (W.X.); 2020022809@m.scnu.edu.cn (W.M.); lisheng@scnu.edu.cn (S.L.); 2 Guangdong Laboratory for Lingnan Modern Agriculture, Genome Analysis Laboratory of the Ministry of Agriculture, Agricultural Genomics Institute at Shenzhen, Chinese Academy of Agricultural Sciences, Shenzhen 518000, China; 3 Guangmeiyuan R&D Center, Guangdong Provincial Key Laboratory of Insect Developmental Biology and Applied Technology, South China Normal University, Meizhou 514779, China

**Keywords:** comparative metabolomics, metabolites, LC–MS/MS, insects

## Abstract

Insect metabolites play vital roles in regulating the physiology, behavior, and numerous adaptations of insects, which has contributed to them becoming the largest class of Animalia. However, systematic metabolomics within the insects is still unclear. The present study performed a widely targeted metabolomics analysis based on the HPLC-MS/MS technology to construct a novel integrated metabolic database presenting comprehensive multimetabolite profiles from nine insect species across three metamorphosis types. A total of 1442 metabolites were identified, including amino acids and their metabolites, organic acids and their derivatives, fatty acids (FAs), glycerophospholipids (GPs), nucleotides and their metabolites, and benzene and its substituted derivatives. Among them, 622 metabolites were used to generate a 0 and 1 matrix based on their presence or absence, and these metabolites were enriched in arachidonic acid metabolism, tyrosine metabolism, phenylalanine metabolism, and insect hormone biosynthesis pathways. Our study revealed that there is a high coincidence between the evolutionary relationships of the species and the hierarchical cluster based on the types of metabolites, while the quantities of the metabolites show a high diversity among species. The metabolome of the nine representative insects provides an important platform for implementing the analysis of insect systemic metabolites and biological events at the metabolic level.

## 1. Introduction

Since their origin ~479 million years ago (Mya), insects, which are extremely diverse and abundant, have become the largest and most evolutionarily successful animal group on earth. Insects have evolved into three different metamorphosis types (ametabola, hemimetabola, and holometabola) and developed numerous metabolites, including hormones, pigments, pheromones, and venom [1,2,3,4,5], that play significant roles in insect growth, development, reproduction, and adaptation to environmental changes [6,7].

Metabolites, known as the “bridge” between genes and phenotypes, are downstream of genetic transcription and translation processes that directly interact with environmental exposures and are thus regarded as being responsible for the phenotype [8,9,10,11]. Metabolomics has been employed to elucidate the key signaling molecules regulating morphological diversity, physiology, and behavior in a variety of aspects of insects [9,11]. For example, changes in metabolites in the mevalonate and juvenile hormone (JH) synthesis pathways in the Corpus allatum (CA) are linked to the reproductive physiology of the mosquito [9]. *Venturia canescens* increases cold tolerance by accumulating metabolites with an assumed cryoprotective function and the depression of metabolites involved in energy metabolism [11]. Compounds associated with energy metabolism were found to deteriorate with age, including hemolymph-rich sugars and large storage glycerolipids in parasitoid wasps [12]. Wu et al. found that carnitine mediates the locust phase transition possibly by modulating lipid metabolism and the nervous system [13]. These studies are confined to one or a certain class of metabolites. The role of multiple insect species of metabolic states in regulating the natural behavior and phenotype of insects is still unclear.

In this study, high-performance liquid chromatography–mass spectrometry (HPLC–MS) and widely targeted metabolomics analysis were used to determine the comprehensive multimetabolite profile of the nine representative insect species covering the three metamorphosis types. Our study indicated that the clustering of the type of metabolites is consistent with the evolutionary relationship of nine species. We also found that the common differential metabolites are likely to evaluate the metabolism of natural species’ physiological variety. We constructed a library of metabolites based on nine insects, which can significantly accelerate the metabolite identification and biological interpretation of other insect species.

## 2. Materials and Methods

### 2.1. Insects

**Figure 1:**
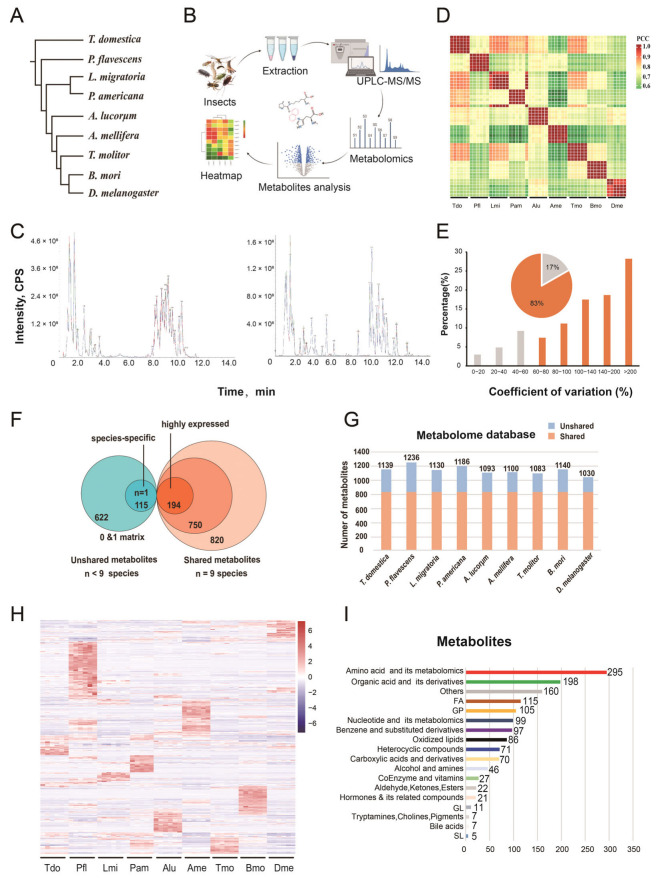

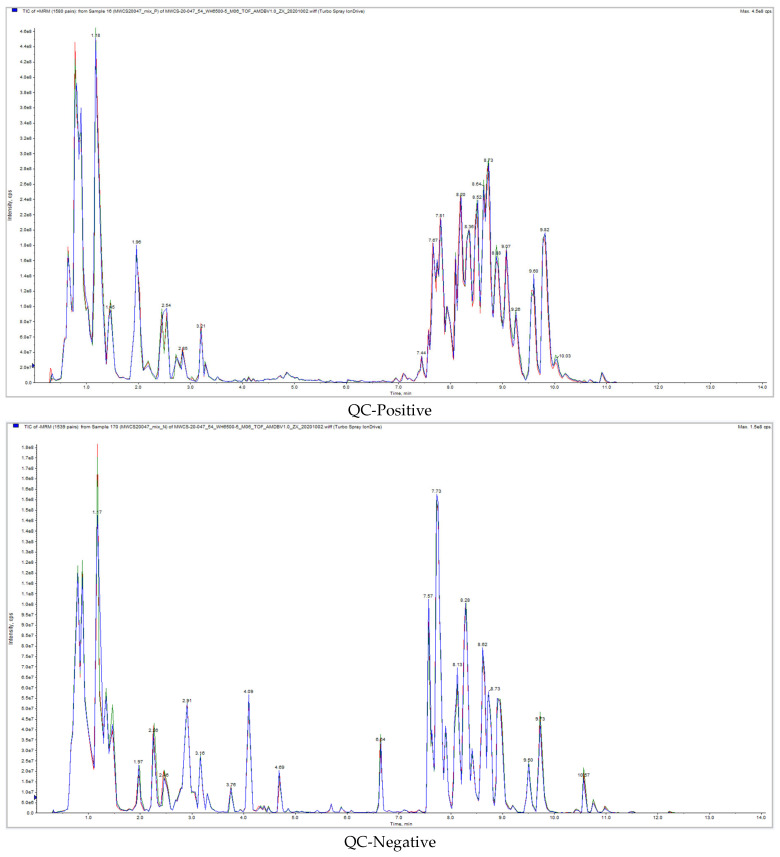
Construction of the metabolome of the nine insect species. (**A**) The nine species were collected for metabolomic analysis, and their phylogenetic tree was generated according to Misof’s publication [14]. These insects include 1 ametabolous type (*T. domestica*), 5 hemimetabolous types (*C. splendens*, *L. migratoria*, *P. americana*, *A. lucorum*, *N. lugens*), and 4 holometabolous types (*A. mellifera*, *T. molitor*, *B. mori*, *D. melanogaster*). (**B**) Widely targeted metabolomics was performed. (**C**) Overlap of the total ion current (TIC) of the quantity control. P+, Positive; P−, Negative. (**D**) The Pearson correlation coefficient (PCC) analysis of 54 samples from 9 species. The number on the left bar indicates the PCCs from 0.6 to 1. (**E**) Coefficients of variation (%). Each species had six replicates. (**F**) The 1442 metabolites identified in the metabolome and their classification. The metabolite information can be found in Table S1. According to their presence or absence in the nine species, the metabolites are classified as unshared (blue, n < 9) or shared types (red, n = 9). Species-specific metabolites (n = 1) represent only one species. (**G**) The metabolites and their classification of 9 species. Red indicates 820 shared metabolites (n = 9) and blue indicates unshared metabolites (n < 9). (**H**) Heatmap of all the metabolites from the nine species. The blue color indicates low content, while the red color indicates high content. (**I**) Annotated metabolite classes.

Nine species representing three metamorphosis types were used in this study, as shown in Figure 1, including one ametabolous species (*Thermobia domestica*), four hemimetabolous species (*Pantala flavecens, Calopteryx splendens, Locusta migratoria, Periplaneta americana, Apolygus lucorum*), and four holometabolous species (*Apis mellifera, Tenebrio molitor*, *Bombyx mori, Drosophila melanogaster*).

The ametabolous group *T. domestica* was reared on paper in the laboratory at 37 °C under a 12:12 h (light:dark) cycle with 75% humidity.

The hemimetabolous groups *P. americana* and *L. migratoria* were raised in the laboratory. The roaches were maintained in a plastic box with enough ddH_2_O and sterile mouse chowing food with 1.06% calcium and 0.99% phosphate under laboratory conditions at 28 °C, 70~80% relative humidity, and a 12:12 h (light:dark) photoperiod; *L. migratoria* collected from Hebei Province was group-reared in large plastic boxes with soil under the bottom at 23 °C and in light during daytime (12 h duration) and ambient temperature (approximately 12 h darkness during night conditions). Insects were fed fresh corn leaves. *A. lucorum* was fed corn at 26 °C, 65% relative humidity, and a 16:8 h (light:dark) photoperiod.

The holometabolous group *D. melanogaster* was grown on standard cornmeal/molasses/yeast food at 25 °C. *T. molitor* was fed wheat bran at 26 °C, 30% relative humidity, and a 12:12 h (light:dark) photoperiod. *A. mellifera* and *B. mori* were gifts from the Institute of Plant Protection at the Chinese Academy of Agricultural Sciences (CAAS), China Agricultural University (CAU), and China Guangdong Academy Agricultural Sciences.

Harvest of insects: To exclude the effect of microorganisms on intestines and foods, we dissected the insects and removed the intestines with a clean scissor and tweezer. The female adults were collected since energy metabolism plays key roles in female reproduction. All 3-day-old female adults were immediately treated with liquid nitrogen and preserved in a −80 °C refrigerator. A total of 54 samples from nine species (6 replicates/species, 10 individuals/replication) were collected for metabolomic analysis. We quantified the whole body of insect metabolites using a broadly targeted liquid chromatography–tandem mass spectrometry (LC–MS/MS)-based metabolic profiling method.

### 2.2. Metabolite Extraction

Metabolite extraction was performed using 70% methanol with an internal target (Dichlorophenylalanine). Briefly, the sample (50 ± 2 mg) was homogenized three times in a ball mill for 3 min at 30 Hz and then ultrasonicated on ice. After adding 1 mL of 70% methanol mixture, whirling for 5 min, and centrifuging at 12,000 rpm for 10 min at 4 °C, the 400 μL supernatant was drawn into a tube and stored at −20 °C overnight. The samples were vortexed for 30 s and centrifuged for 3 min at 12,000 rpm at 4 °C. A total of 200 μL of the supernatant was transferred to the liner of the corresponding injection bottle for on-board analysis. Equal parts from each sample were pooled as QC samples.

### 2.3. UPLC Conditions

The extracts were analyzed using a LC–ESI–MS/MS Method Conditions System (UPLC, Shim-pack UFLC SHIMADZU CBM A system, SHIMADZU, Guangzhou, China; MS, QTRAP^®^ System, SCIEX, Framingham, MA, USA). An ACQUITY UPLC HSS T3 C18 column (1.8 μm, 2.1 mm × 100 mm) was collected, and the column temperature was maintained at 4 °C with a 0.4 mL/min flow rate, 2 μL/5 μL injection, and solvent system of water (0.1% formic acid):acetonitrile (0.1% formic acid). The gradient program was as follows: 95:5 *v*/*v* at 0 min, 10:90 *v*/*v* at 10.0 min, 10:90 *v*/*v* at 11.0 min, 95:5 *v*/*v* at 11.1 min, and 95:5 *v*/*v* at 14.0 min.

### 2.4. QTOF-MS/MS

Mass spectrometric data were obtained on an information-dependent analysis (IDA) by the acquisition software (TripleTOF 6600, AB SCIEX). In each cycle, 12 precursor ions (intensity > 100) were chosen for fragmentation at a collision energy (CE) of 30 V (12 MS/MS events with a product ion accumulation time of 50 msec each). ESI source conditions: ion source gas 1, 50 psi; ion source gas 2, 50 psi; curtain gas, 25 psi; source temperature, 500 (positive); and ion spray voltage floating (ISVF), 5500 V or −4500 V (negative).

### 2.5. ESI-Q TRAP-MS/MS

LIT and triple-quadrupole (QQQ) scans were obtained from a triple-quadrupole linear-ion-trap mass spectrometer (QTRAP), QTRAP^®^LC–MS/MS System, and an ESI Turbo Ion-Spray interface. This instrument was operated in positive and negative ion mode with Analyst 1.6.3 software (Sciex). The ESI source was set as follows: drying gas temperature, 500 °C; ion spray voltage (IS), 5500 V (positive), −4500 V (negative); ion source gas I (GSI), 50 psi; gas II (GSII), 50 psi; curtain gas (CUR), 25 psi; and high collision gas (CAD). Instrument tuning and mass calibration were carried out with 10 and 100 μmol/L polypropylene glycol solutions in QQQ and LIT modes, respectively. Based on the metabolites eluted within this period, a set of MRM transitions were managed for each period.

### 2.6. PCA

Unsupervised PCA (principal component analysis) was performed by the statistics function prcomp within R (www.r-project.org) (accessed on 23 January 2023). The data were unit-variance-scaled before unsupervised PCA. To visualize the differences between groups, the unsupervised dimensionality reduction method of principal component analysis (PCA) was applied in all samples using R package models (http://www.r-project.org/ accessed on 23 January 2023). PCA is a statistical procedure that converts hundreds of thousands of correlated metabolite variables into a set of values of linearly uncorrelated variables called principal components.

### 2.7. Hierarchical Cluster Analysis and Pearson Correlation Coefficients

The HCA (hierarchical cluster analysis) results between samples were shown as heatmaps with dendrograms. Pearson correlation coefficients (PCCs) were calculated by the core function in R and presented as heatmaps. Both HCA and PCC were conducted by the R package Complex Heatmap. For HCA, normalized signal intensities of metabolites (unit variance scaling) were visualized with a color spectrum.

### 2.8. Differential Metabolite Analysis

Significantly regulated metabolites were obtained based on VIP value and fold change (VIP ≥ 1 and absolute Log2FC (fold change ≥ 1). VIP values were from the OPLS-DA results with score plots and permutation plots, which were generated by using the R package MetaboAnalystR. The data were log-transformed (log2) and mean-centered before OPLS-DA. To avoid overfitting, a permutation test (200 permutations) was performed.

### 2.9. KEGG Pathway Analysis

The metabolites were identified and annotated according to the KEGG Compound database (http://www.kegg.jp/kegg/compound/ accessed on 23 January 2023) and the KEGG Pathway database (http://www.kegg.jp/kegg/pathway.html accessed on 23 January 2023). Significantly enriched metabolic pathways in differential metabolites were compared with the whole background signal. The data were analyzed by Metabolite Set Enrichment Analysis (MSEA; https://rdrr.io/github/afukushima/MSEAp/ accessed on 23 January 2023). Pathways mapped with significantly regulated metabolites were then fed into MSEA (metabolite set enrichment analysis), and their significance was determined by hypergeometric test *p* values.

## 3. Results

### 3.1. Construction of a Metabolic Dataset

To better understand the functions of the metabolites during insect evolution, we selected 9 species (54 samples, six replications/species) from the three types of metamorphosis in insects (Figure 1A) [14]. Wide-targeted metabolomics of 54 samples was carried out, and all metabolite peaks were assigned by a secondary mass spectrometry database (Figure 1B). The metabolic profiles of samples with quality control (mix of all the samples) were acquired by the validated UPLC–MS methods in positive and negative ion modes, and further analysis indicated the high reliability of the data (Figure 1C). The correlation of six replications of each species indicated a strong correlation (Figure 1D). The coefficients of variation (CVs) of more than 82.94% of metabolites were greater than 60%, indicating obvious differences among the metabolites (Figure 1E).

### 3.2. The Identified Metabolites of Nine Species for the Global Overview

A total of 1442 components from nine species were tentatively identified by comparing the GC–MS data with the insect metabolic database to obtain a metabolome data matrix (Table S1, Figure 1F). The nine species shared 820 metabolites, and 750 of them were defined as common metabolites on the basis of the threshold value. The other 622 metabolites detected in fewer than nine species were defined as unshared compounds in blue (Figure 1F). The number of metabolites for each species is shown in Figure 1G. The total amount of metabolites for most species was approximately 1100, except for *P. flavescens* (1236) and *D. melanogaster* (1030). The heatmap showed that six replicates of each species were closely clustered together, illustrating the reliability and similarity between the replications (Figure 1H). Interestingly, each species had its own relatively highly expressed metabolite pattern, especially *P. flavescens*. All the metabolites were clustered into 18 categories (Figure 1I). Approximately 50% of metabolites were enriched in amino acids and their metabolites (295, 21%), organic acids and their derivatives (198, 14%), and unknown other metabolites (160, 11%), representing the fundamental and essential metabolic process in the insects. The other groups were fatty acids (FAs) (115, 8%), glyceryl phosphatides (GPs) (105, 7%), nucleotides and their metabolites, and benzene and its substituted derivatives.

**Figure 2:**
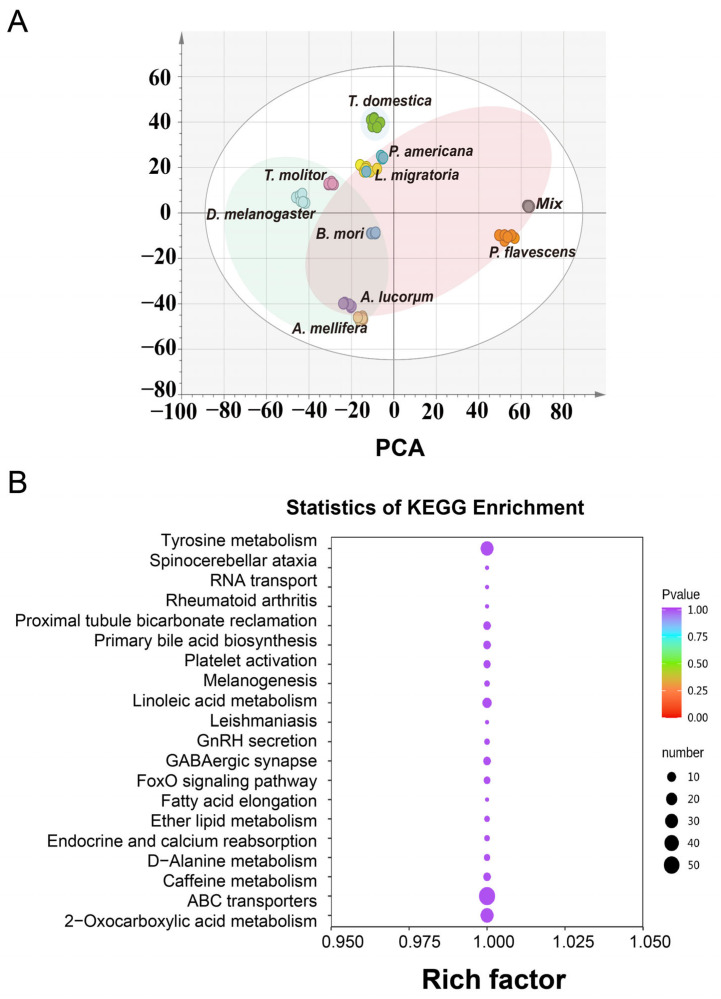
Global analysis of the metabolites of nine species. (**A**) PCA of all the samples. (**B**) KEGG enrichment of all the metabolite-matched metabolic pathways.

The results of each species (6 replications/species) were clustered using PCA (Figure 2A) and were consistent with the heatmap results (Figure 1H). The PCA results revealed a discrimination between *P. flavescens* and other species. *A. lucorum* also showed an unexpected distance from the other hemimetamorphosis species. *T. domestica* was closer to the two hemimetamorphosis species (*P. americana* and *L. migratoria*), and four species from the holometabolous type were clustered together, which represents the basal evolution status of the three metamorphosis types. The three most enriched pathways of the metabolites based on KEGG analysis were related to ATP-binding cassette (ABC) transporters, tyrosine metabolism, and 2-oxocarboxylic acid metabolism (Figure 2B).

**Figure 3:**
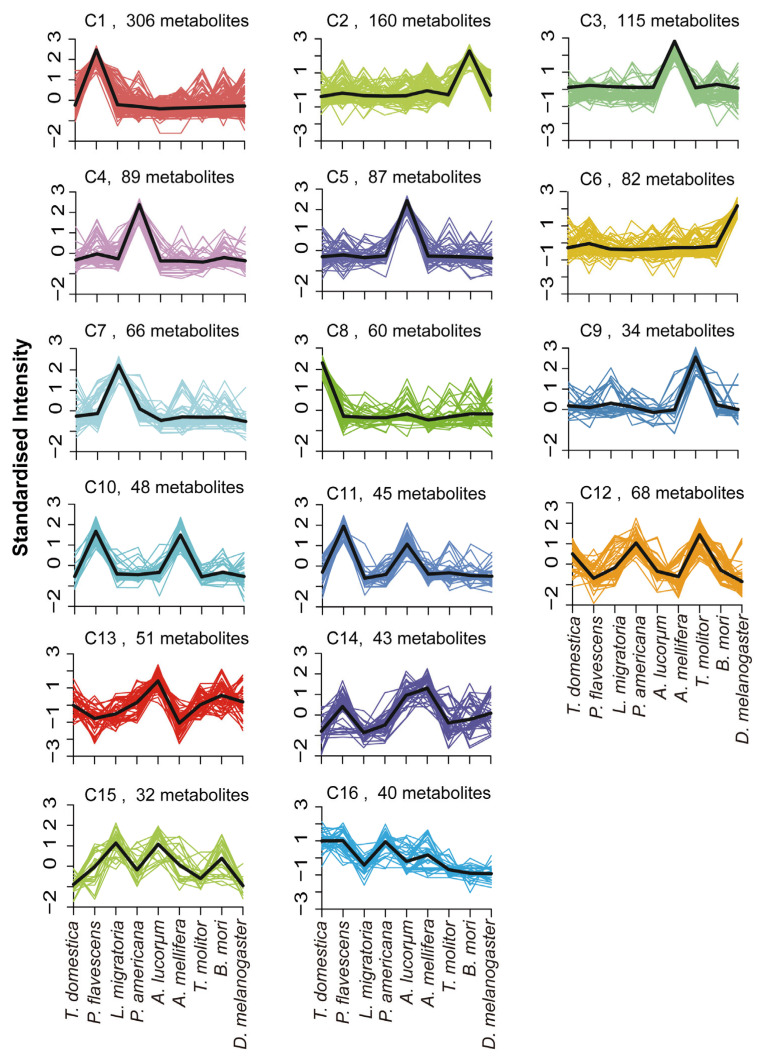
Differential expression patterns of the 16 subclasses of metabolites. Based on the expression pattern, the metabolites were divided into the 16 classes. The single peak means that these metabolites were highly expressed in one species.

**Figure 4:**
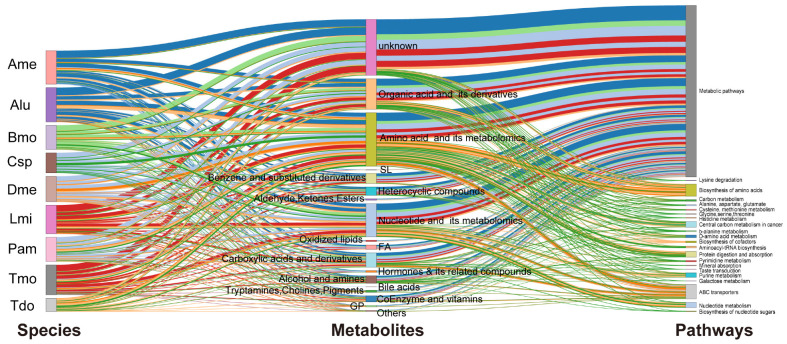
A Sankey diagram delineating the links between insect species, metabolites, and the top enriched pathways. The Sankey diagram is used for depicting the distribution and fate of the metabolites from nine species. Thickness is proportional to the contribution of a given pathway or class.

To reveal the changes in differential metabolites among the species, we first performed centralization and standardization of the relative content of metabolites. Then, we used K-means clustering, involving calculating the distance measure for all values, and created a new center-based point that represented the means of values for each cluster. These clusters represented the distribution of differential metabolites in the nine species. As shown in Figure 3, the metabolites were divided into 16 metabolite subclasses. Surprisingly, there were nine subclasses indicating species-specific, highly expressed metabolite patterns, including Sub Class 9 (306 metabolites with high content in *P. flavescens*)*,* Sub Class 15 (160 metabolites with high content in *B. mori*), and Sub Class 5 (115 metabolites with high content in *A. mellifera*). For the three metamorphosis groups, 60 metabolites (subclass 8) were highly expressed only in *T. domestica*. Each species possessed its own highly expressed metabolites, consistent with the heatmap (Figure 1H). There were 70% (Class 1 to 9) of metabolites with higher content in one species. The numbers of metabolites with higher abundance in two or more species were 93 and 234. The majority of metabolites belonged to metabolic pathways, ABC transporters, and the biosynthesis of amino acids (Figure 4). These data showed that the dispersion among the species may depend not on the quantity but on the quantity of the metabolites. In addition, the differential metabolites of one species were compared to each of the other eight species to obtain eight groups. The intersection among the eight groups was selected to generate the specific metabolites’ expression pattern in one species (Figure S1). As shown in Figure S1, each of the nine species possessed their own specific metabolites. Taken together, these results revealed that the species-specific metabolites play a key role in the species cluster. 

### 3.3. Common Metabolic Enrichments and Pathways

**Figure 5:**
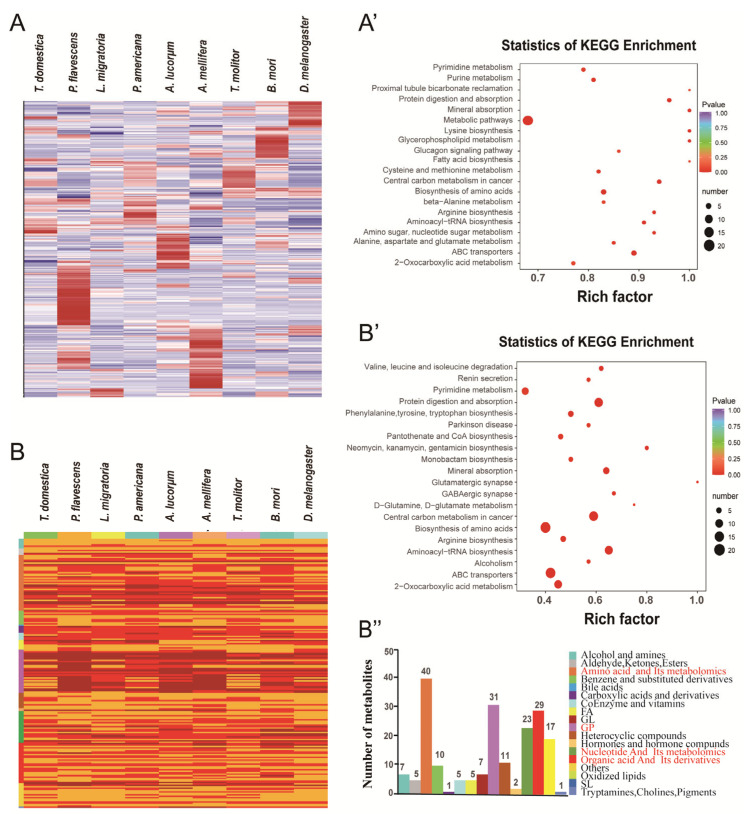
The common metabolites in all the insects. Heatmap (**A**) and KEGG enrichment (**A’**) of the 70 common metabolites. Heatmap (**B**), KEGG enrichment (**B’**), and metabolites (**B’’**) of the 194 highly expressed metabolites. The ordinate shows the relative content of different classes of metabolites.

A total of 750 common expressed metabolites was found among the nine species, and the largest number of identified metabolites were involved in the metabolic pathway (Figure 5A,A’). Among them, 194 (value > 10^6^) metabolites were highly expressed in all species and were enriched in five key pathways: amino acids and their metabolites (40, 21%), GPs (31, 16%), organic acids and their metabolites (29, 15%), nucleotides and their metabolites (23, 12%), and hormones and hormone compounds (17, 9%) (Figure 5B–B’’). The results showed that ABC transporters, the biosynthesis of amino acids, central carbon metabolism in cancer metabolism, protein digestion and absorption, aminoacyl-tRNA biosynthesis, and 2-oxocarboxylic acid metabolism were the common pathways enriched in the insects.

### 3.4. Comparison of the Metabolites between Hemimetabolous and Holometabolous Insects

**Figure 6:**
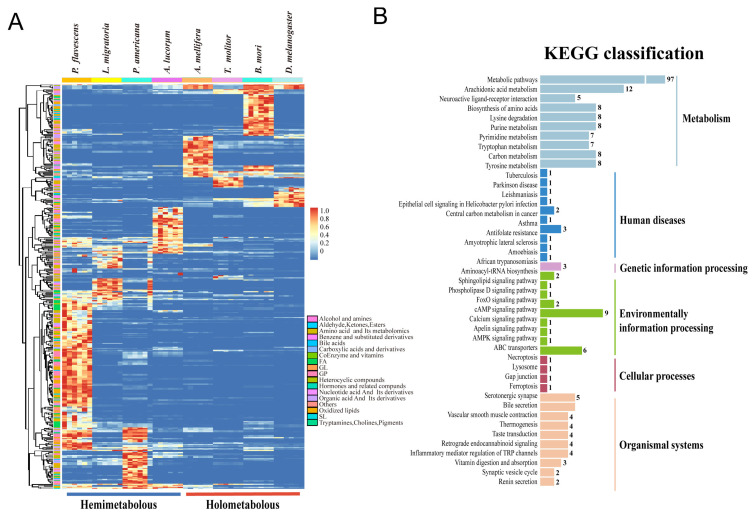
Analysis of the differential metabolites in the species. (**A**) Heatmap of the metabolites in the two metamorphosis types (n < 9) and (**B**) its classification of KEGG enrichment.

Comparison of the metabolites between hemimetabolous and holometabolous types revealed that each species has its own specific metabolites, particularly highly expressed metabolites (Figure 6A). For example, *P. flavecens* showed the richest highly expressed metabolites, followed by *B. mori*, which contained many amino acids, acids, and lipids. The least abundant species was *T. molitor*, which contained several amino acids. These metabolites were clustered into two large groups: the upper holometabolous type and the lower hemimetabolous type. Overall, in comparison to the holometabolous species, there were more abundant upregulated metabolites observed in the hemimetabolous types. The metabolites of eight species were categorized into four key classes. First, the highly abundant metabolites from *P. flavecens* and *P. americana* were clustered, and then they were grouped with *L. migratoria* and A. lucorum. Finally, *D. melanogaster* and the other three species were clustered together, implying that there are individually conserved or basal metabolic pathways for each metamorphosis type. Furthermore, KEGG analysis showed that these differential metabolites were enriched in at least six groups, namely, metabolism (168), organism systems (37), environmental information processing (24), human diseases (13), genetic information processing (3), and cellular processes (4), suggesting that most of these metabolites are basal and conserved within and among the animals.

### 3.5. Quantitative Statistics of Significantly Changed Metabolites between the Species of Interest

**Figure 7:**
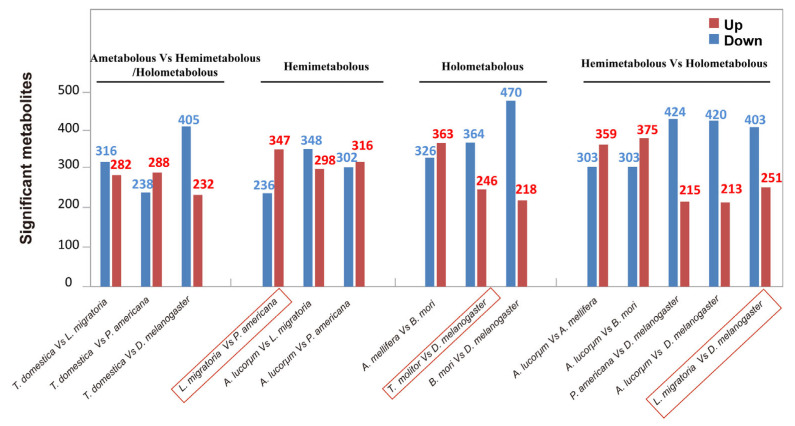
The differential metabolites between the species of interest.

**Figure 8:**
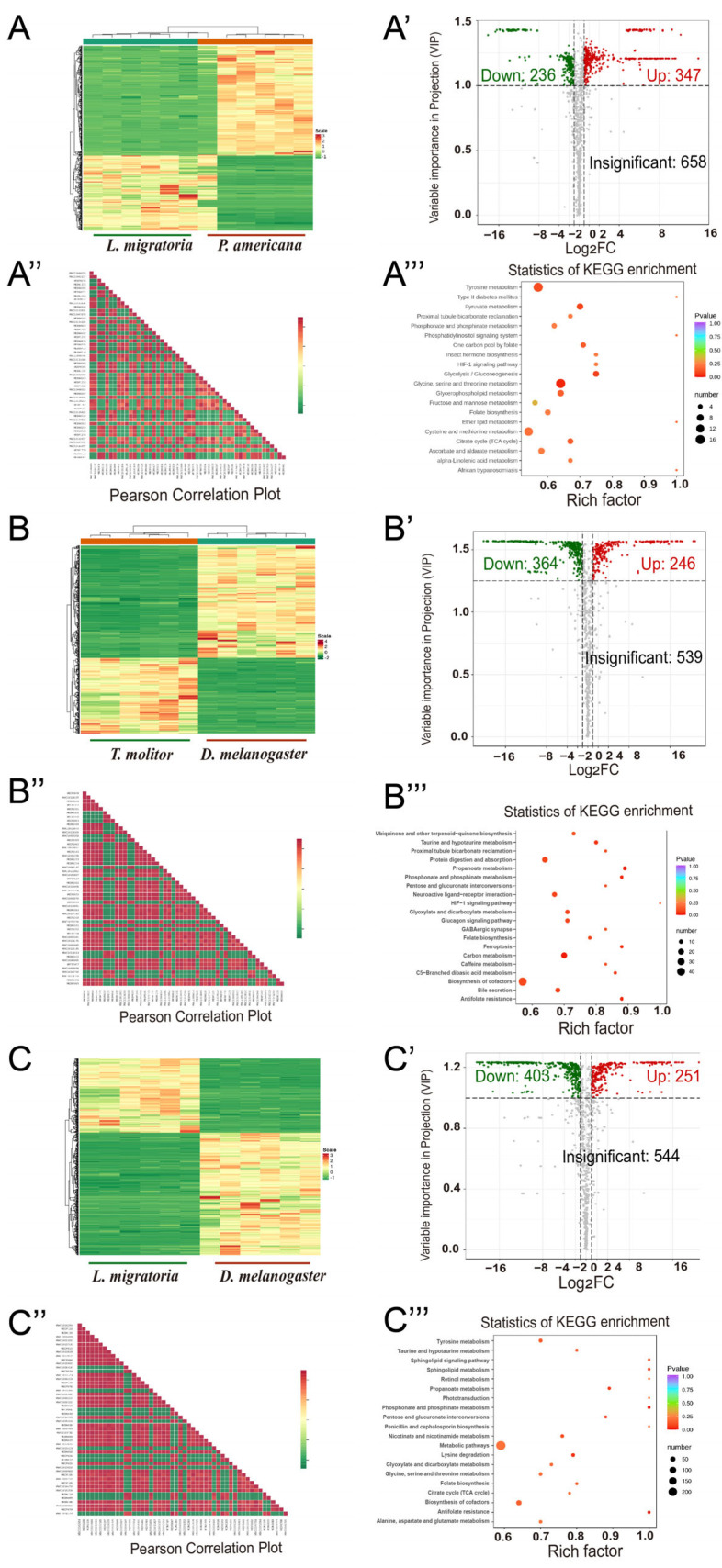
Heatmap, volcano plot, Pearson correlation plot, and KEGG enrichment of the metabolites between *L. migratoria* and *P. americana* (**A**−**A’’’**), *T. molitor* and *D. melanogaster* (**B**−**B’’’**), and *L. migratoria* and *D. melanogaster* (**C**−**C’’’**).

High-throughput metabolomics allowed for us to compare the metabolic profiles between the two species, and the common differential metabolites produced in the different groups were analyzed (Figure 6, Figure 7 and Figure 8 and Figures S2–S6; Table S2). Within the hemimetabolous type, there were 583 differential metabolites in *L. migratoria* vs. *P. americana*, 646 in *A. lucorum* vs. *L. migratoria*, and 618 in *A. lucorum* vs. *P. americana*. In the *L. migratoria* vs. *P. americana* comparison group, the contents of 236 metabolites significantly decreased, while those of 347 metabolites notably increased (Figure 8A–A’’). Within the holometabolous type, there were 610 differential metabolites in *T. molitor* vs. *D. melanogaster*, 689 in *A. mellifera* vs. *B. mori*, and 688 in *B. mori* vs. *D. melanogaster*. In the *T. molitor* vs. *D. melanogaster* comparison group, 364 metabolites were upregulated, while 246 metabolites were downregulated (Figure 8C–C’’). Similarly, there were fewer total differential metabolites (654,403 upregulated and 251 downregulated) between *L. migratoria* and *D. melanogaster* than between the other pairs (Figure 8E–E’’). In comparison to *B. mori*, there were 470 downregulated and 218 upregulated metabolites in *D. melanogaster* (Figure 7). More upregulated metabolites in other species showed reduced levels in *D. melanogaster*. Taken together, these data indicate that under the same metamorphosis type, the fewer differential metabolites there are, the closer their relationship is at the metabolic level.

### 3.6. The Quality of Metabolites Contributes to the Cluster Relationship of Insects

**Figure 9:**
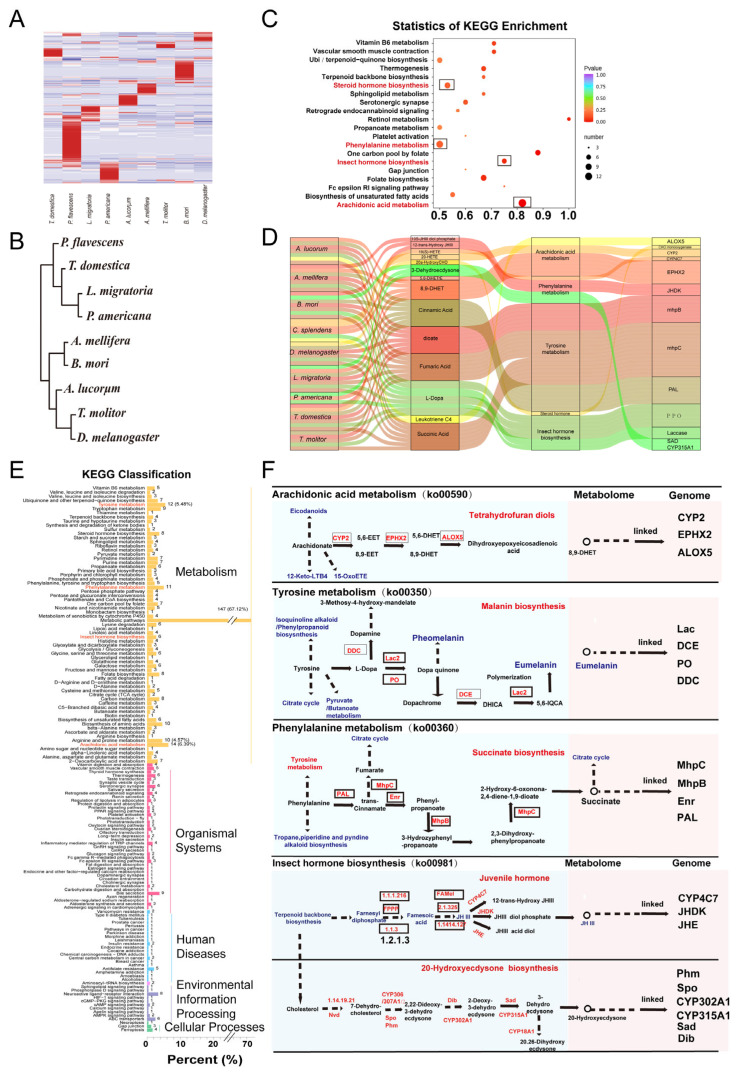
Qualitative analysis of metabolites (622) in the nine species. (**A**) Heatmap of these metabolites expressed in the species (n < 9). (**B**) Cluster analysis of the metabolites based on a 0/1 matrix. (**C**) KEGG enrichment of the metabolite-matched metabolic pathways. (**D**) A Sankey diagram delineating the links between insect species, metabolites, top enriched pathways, and inferred genes/proteins. (**E**) KEGG classification of the metabolites. (**F**) The top KEGG pathways and associated proteins.

The quantity of metabolites varied among species, growth stages of insects, and environmental factors. To clearly elucidate the quantitative correlation between metabolites and the actual evolutionary relationship among the species, the metabolome matrix (0/1) of 622 metabolites (Figure 9A) was subjected to hierarchical cluster analysis. The results showed that these metabolites were discriminated into two clusters that generally represent the three metamorphosis types. First, there was a relatively close relationship between *L. migratoria* and *P. americana,* and *T. domestica* and *P. flavescens* were clustered together. Another cluster included *D. melanogaster* and *T. monitor*, *A. lucorum*, *A. mellifera,* and *B. mori* (Figure 9B). These data revealed that there is a high coincidence between the actual evolution relationship of the species and the hierarchical cluster on the basis of the qualitative analysis of the metabolites (Figure 1A).

KEGG analysis showed that these metabolites were mostly enriched in metabolic pathways (67%), including arachidonic acid metabolism, tyrosine metabolism, phenylalanine metabolism, and insect hormone biosynthesis (Figure 9C). A number of chemical compounds were identified, including dopa, dopa quinone, dopachrome, 8, 9-dihydroxyeicosatrienoic acid (8,9-DHET), and succinate. Notably, a number of proteins were linked, including cytochrome P450s (CYPs), laccase (Lac), and hormone-related proteins (Figure 9D–F). Among the metabolites related to tyrosine and phenylalanine metabolism, we found that dopa and dopa quinone played important roles in the development of adults, especially eumelanin deposition. We also deduced that insect hormone biosynthesis (juvenile hormone, JH; 20-hydroxyecdysone, 20E) is essential and abundant during the development and metamorphosis of insects.

**Figure 10:**
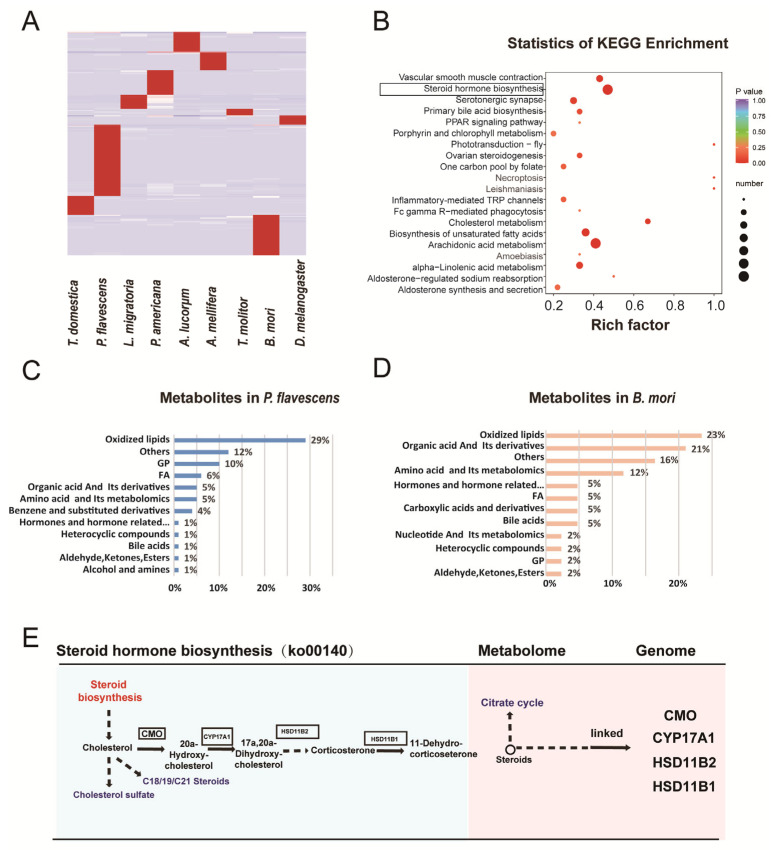
Analysis of the species-specific metabolites. (**A**) Heatmap of the 115 species-specific metabolites. (**B**) KEGG enrichment of the specific metabolites. (**C**) Specific metabolites in *P. flavescens*. (**D**) Specific metabolites in *B. mori*. (**E**) The top steroid hormone biosynthesis pathway.

We then compared 115 species-specific metabolites that were enriched in steroid hormone biosynthesis, arachidonic acid metabolism, biosynthesis of unsaturated fatty acids, etc. (Figure 10A,B). Among the nine species, *P. flavecens* and *B. mori* are much more abundant than the other species. *P. flavecens* needs more energy to fly than some other insect species, and the energy sources include oxidized lipids, GPs, and FAs. *B. mori* stores more energy for reproduction, including oxidized lipids, organic acids and their derivatives, amino acids, and metabolites (Figure 10C–E). Oxidized lipids are very important energy sources of the cell, and energy metabolism may play an important role during adult development. *P. flavescens* and *B. mori* contained the most species-specific metabolites. The specific metabolites are mainly oxidized lipids (for example, eicosapentaenoic acid) and glycerophospholipids in *P. flavescens*, which are the preferred fuel for their strong flight ability [15]. A relatively high amount of glycerides in female adult silkworms may be key energy for reproduction since silkworms store plenty of lipids and cannot fly, as a result of thousands of domestications. Lipids are the main energy source for the development of embryos.

Taken together, we conclude that the genetic evolution in insects can be explained by the quality of the metabolites. Species-specific metabolites had the highest contribution to the cluster that played key roles during insect evolution at the metabolic level. These metabolites from female individuals will provide significant benefits for animal production.

## 4. Discussion

Insect metabolites play vital roles in regulating the behavior of herbivorous insects. The metabolome is a desirable tool to predict the phenotype of an organism to gene genetic alterations or environmental influences [16,17]. In this study, we revealed the overall metabolic profile of the insects from nine species with LC–MS/MS-based metabolomics. 

Our metabolome revealed that the 1442 metabolites (amino acids, nucleotides, fatty acids, organic acids, etc.) with 43 related pathways are involved in ABC transporter systems, tyrosine metabolism, 2-oxocarboxylic acid metabolism, etc. Amino acids and their metabolites are integrated molecules of functional and structural proteins. Organic acids and their derivatives widely occur in animal, plant, and microbial sources. Fatty acids play many essential roles in insects, including the provision of energy for the blood and fat body [18]. ABC transporters, as exporters or importers, are involved in the multiple ATP-powered translocation of many substrates across membranes extracellularly and intracellularly, such as amino acids, nucleotides, carbohydrates, lipid-related compounds, and vitamins [19,20,21]. 2-Oxocarboxylic acids are the most elementary set of many metabolites (pyruvate, 2-oxobutanoate, oxaloacetate, and 2-oxoglutarate) and are related to other metabolic pathways, such as the tricarboxylic pathway and amino acid synthesis pathway [22]. Thus, these compounds are responsible for a set of biochemical transformations in living cells to maintain the growth, development, metamorphosis, and immune responses of insects.

We originally tried to construct the phylogenetic tree based on the quantity of all the metabolites and we did not obtain the expected results. The number of compounds with divergent physical properties is generally subjected to significant environmental factors (temperature, flight ability, etc.). We therefore applied a 0 and 1 matrix to analyze the distance within the species. Interestingly, the constructed cluster from unique metabolites (622) highly matches their natural relationship (Figure 1A and Figure 9B). Thus, we draw a conclusion that the unique metabolites among the species coincide with the actual separation based on the current genetic relationship (Figure 9). These metabolites are enriched in arachidonic acid (ARA) metabolism, tyrosine metabolism, phenylalanine metabolism, insect hormone biosynthesis, etc. Additionally, these proteins in the pathways are involved in insect-immunity-related enzymes, detoxification enzymes (CYP2), melamine (Lac), and insect hormones (CYP15A1, JHAMT). Free arachidonic acid serves as the substrate for cyclooxygenases, lipoxygenases, and cytochromes P-450 (CYPs). The ARA metabolism pathway generates diverse, fast-acting biologically active molecules (lipids, 17-HETE, etc.) to modulate insect immunity [21]. Phenylalanine and tyrosine metabolism are involved in the early steps of the sclerotization and melanization of cuticles [22,23]. In brief, phenylalanine is first dehydroxylated to tyrosine. Tyrosine is catalytically converted to a variety of quinones and melanochromes, causing melanization. As a critical endocrine regulator, JH regulates multiple physiological processes in insect adults, including molting, metamorphosis, growth, reproduction, and oviposition. Juvenile hormone acid methyltransferase (JHAMT) and methyl farnesoate epoxidase (CYP15A1) are key rate-limiting enzymes during JH biosynthesis in the corpus allatum (CA). JH regulates fat body vitelline (Vg) synthesis and aids in ovary maturation in *Locusta migratoria*, *Aedes aegypti* and *Drosophila* [24,25].

Biological metabolites are sensitive and susceptible to changes with the external or internal environment, including the genotype of the animal, its diet, environment, inherited epigenetic effects, circadian rhythms, and the gut microbiome. Each of these factors, alone or in combination, can have a profound effect on metabolite levels, leading to different overall results. Individual traits of life history and metabolism remain unresolved. Dispersal capability drives the evolution of metabolic capacities in *Gryllus firmus* [26]. Flight capability is associated with larger in vivo metabolic capacities. Typically, dragonflies have strong flight muscles to metabolize lipids, and they engage in two types of flights: brief and sustained flights. The initial brief flights are rapidly fueled by carbohydrates (sugars), and then during the prolonged flights, insect muscles perform a transition to lipid oxidation (fats) [27,28]. Consistent with our results, *P. flavescens* with more oxidized lipids showed a remote relationship with the others (Figure 2A and Figure 10C). In addition, the metabolites change dramatically from the early to late stage of *Drosophila* embryogenesis [29]. In *Drosophila*, the network structure of metabolites is altered upon dietary restriction and plays an important role in preventing the decline in homeostasis with age [30]. Thus, further studies should be performed on tissue-specific metabolites or the species under the same treatment. Although metabolism reveals multiple differential metabolites and pathways, to analyze the molecular regulation among the metabolites, further study should integrate multiple omics (transcriptomics or proteomics) to analyze the molecular regulation of certain metabolites under stress factors or metabolome-assisted plant or animal biology [1,31,32,33,34,35].

Our study revealed that there is a high coincidence between the evolutionary relationships of the species and the hierarchical cluster based on the types of metabolites, while the quantities of the metabolites show a high diversity among species. The metabolome of multiple species enhances the current understanding of the biological functions of metabolites in insects, which is a crucial platform to uncover hidden associations between genetic and environmental components.

## Data Availability

Data is contained within the supplementary material.

## Supplementary Materials

The following supporting information can be downloaded at: https://www.mdpi.com/article/10.3390/metabo13060735/s1, Figure S1: The species-specific differential metabolites among the species; Figures S2–S6: The differential metabolites between the interested species; Table S1: All the metabolites (1442) identified in insects.
