# Supplementary material for: Widely Targeted HPLC-MS/MS Metabolomics Analysis Reveals Natural Metabolic Insights in Insects
Source: Metabolites. 2023 Jun 8;13(6):735. doi: 10.3390/metabo13060735 (PMC10301993; doi:10.3390/metabo13060735)
Supplement: Supplementary file 1 [file metabolites-13-00735-s001.zip › metabolites-2372872-supplementary.pdf]

metabolites

Supplementary Materials for

Widely Targeted HPLC-MS/MS Metabolomics Analysis Reveals Natural Metabolic Insights in Insects

Zhaoxin Li [1,2,3,] Yunlong Cheng [1], Jinxin Chen [1], Weijun Xu [1], Wentao Ma [1], Sheng Li [1,2,3], and Erxia Du [1,2,3,*]

Supplementary materials:

Figure. S1 to S6

Table S1

Figures

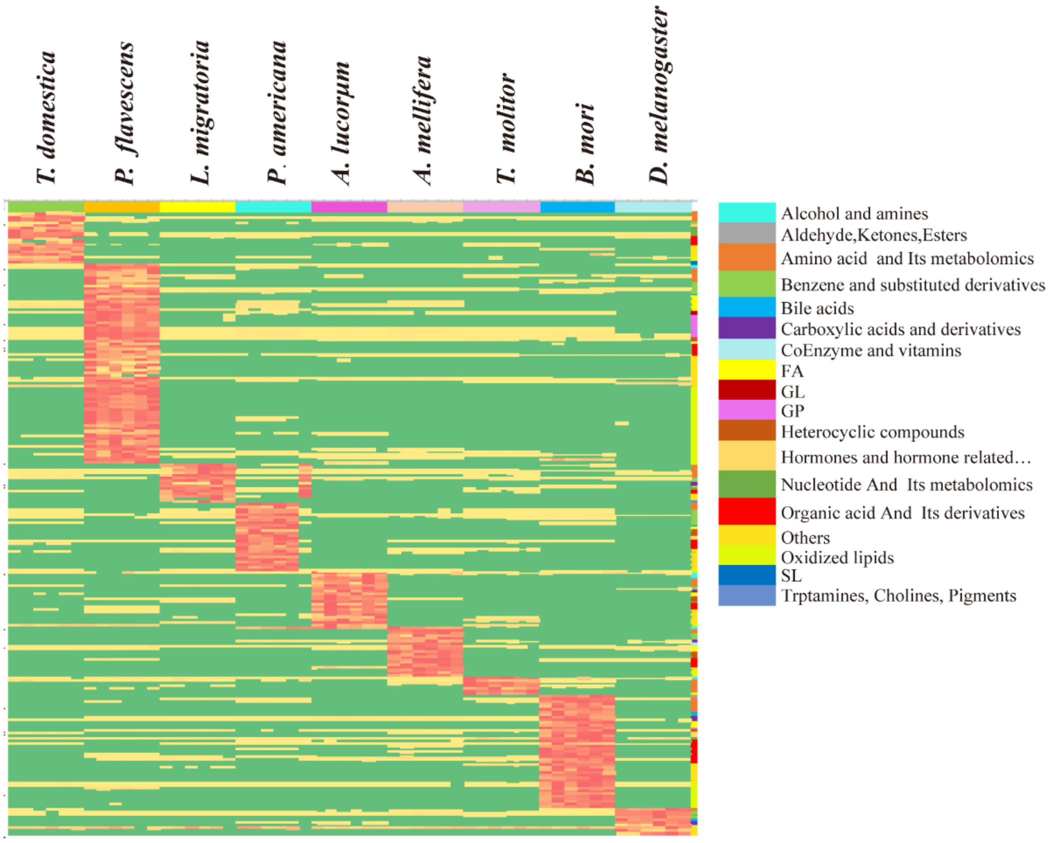

Figure S1. The species-specific differential metabolites among the species. The differential metabolites of one species was compared to each of the other 8 species to obtain 8 groups. The intersection among the 8 groups were selected to generate the specific metablites expression pattern in one species.

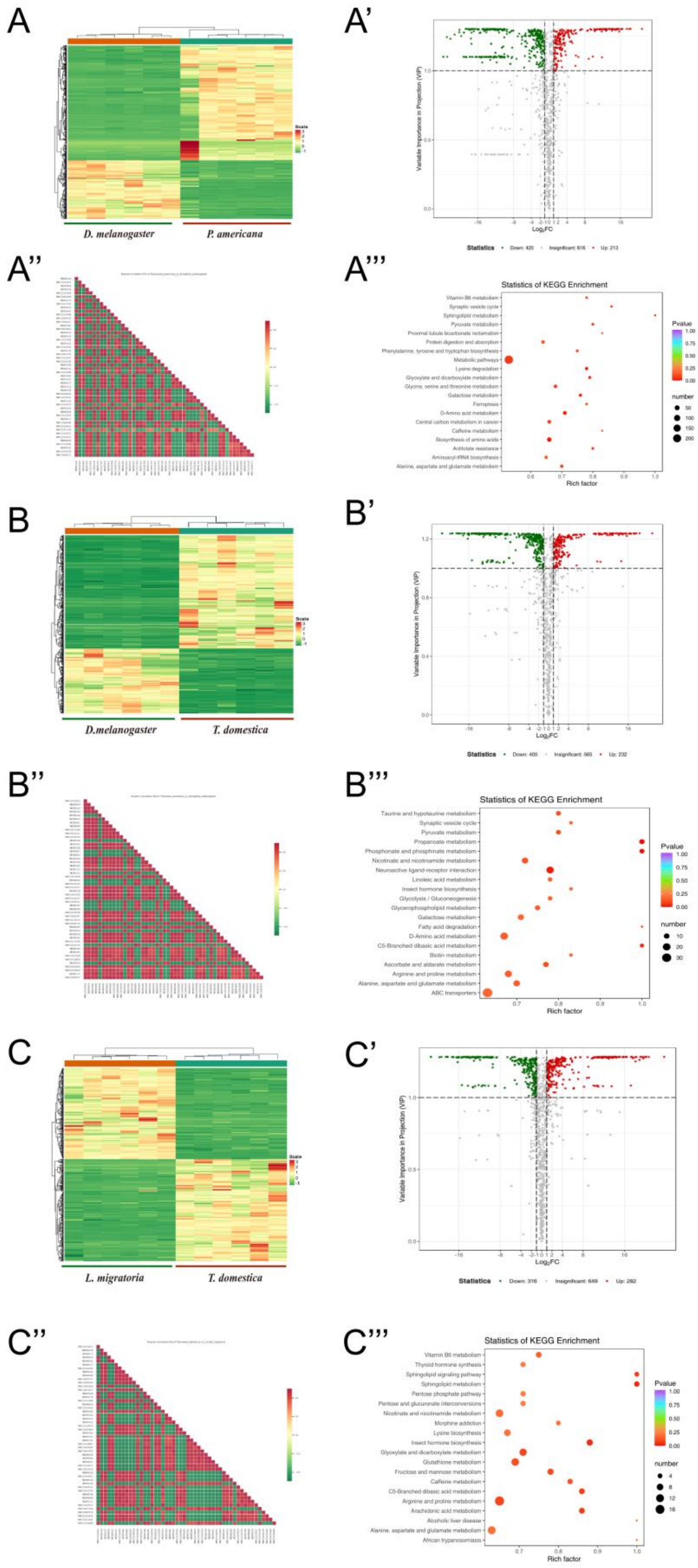

Figure S2. Heatmap, volcano plot, Pearson correlation plot, and KEGG enrichment of the metabolites between *D. melanogaster* vs *P. americana* **(A-A''')**, *D. melanogaster* vs *T. domestica* **(B-B''')**, *L. migratoria* vs *T. domestica* **(C-C''')**.

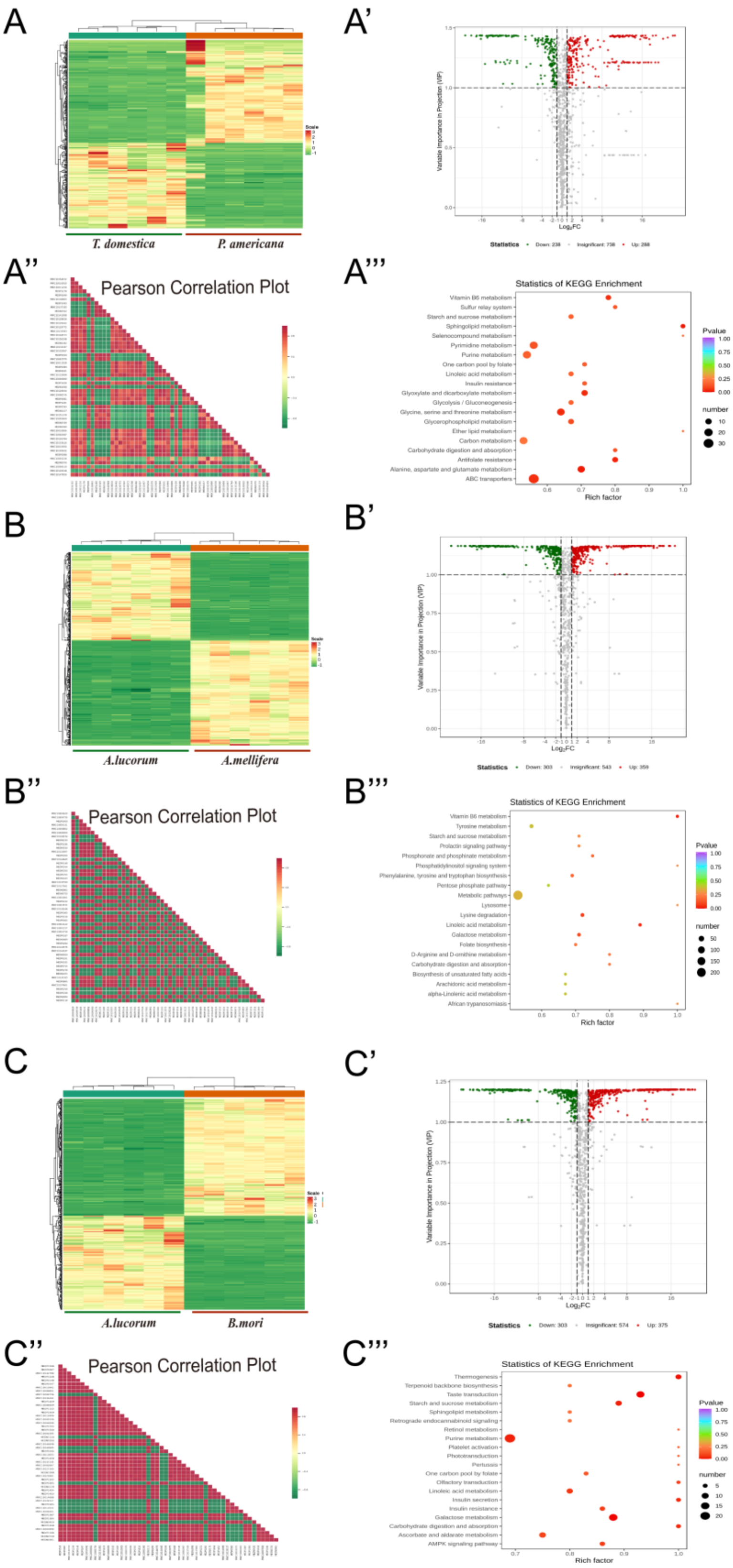

Figure S3. Heatmap, volcano plot, Pearson correlation plot, and KEGG enrichment of the metabolites between *T. domestica* vs *P. americana* **(A-A''')**, *A. lucorum* vs *A. mellifera* **(B-B''')**, *A. lucorum* vs *B. mori* **(C-C''')**.

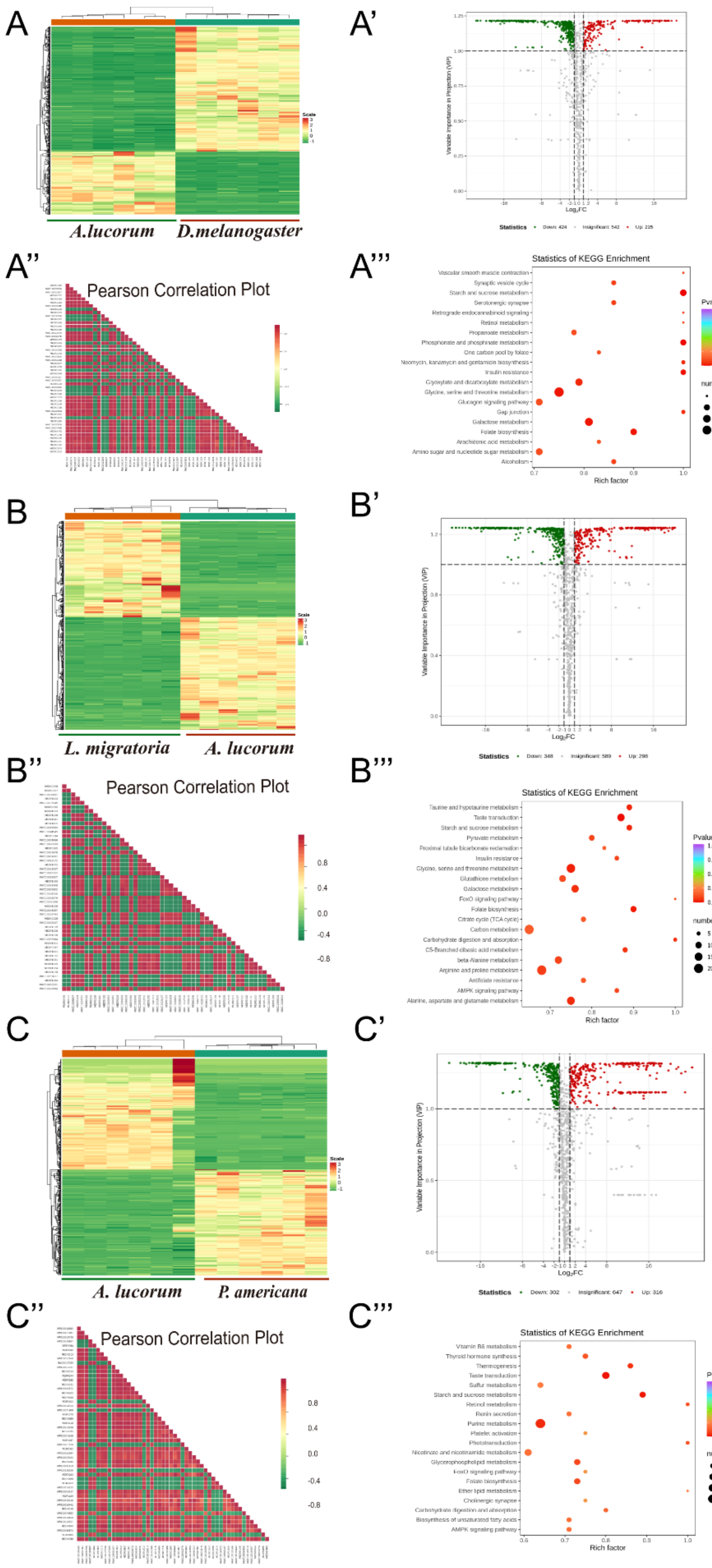

Figure S4. Heatmap, volcano plot, Pearson correlation plot, and KEGG enrichment of the metabolites between *A. lucorum* vs *D. melanogaster* **(A-A''')**. *L. migratoria* vs *A. lucorum* **(B-B''')**, *A. lucorum* vs *P. americana* **(C-C''')**.

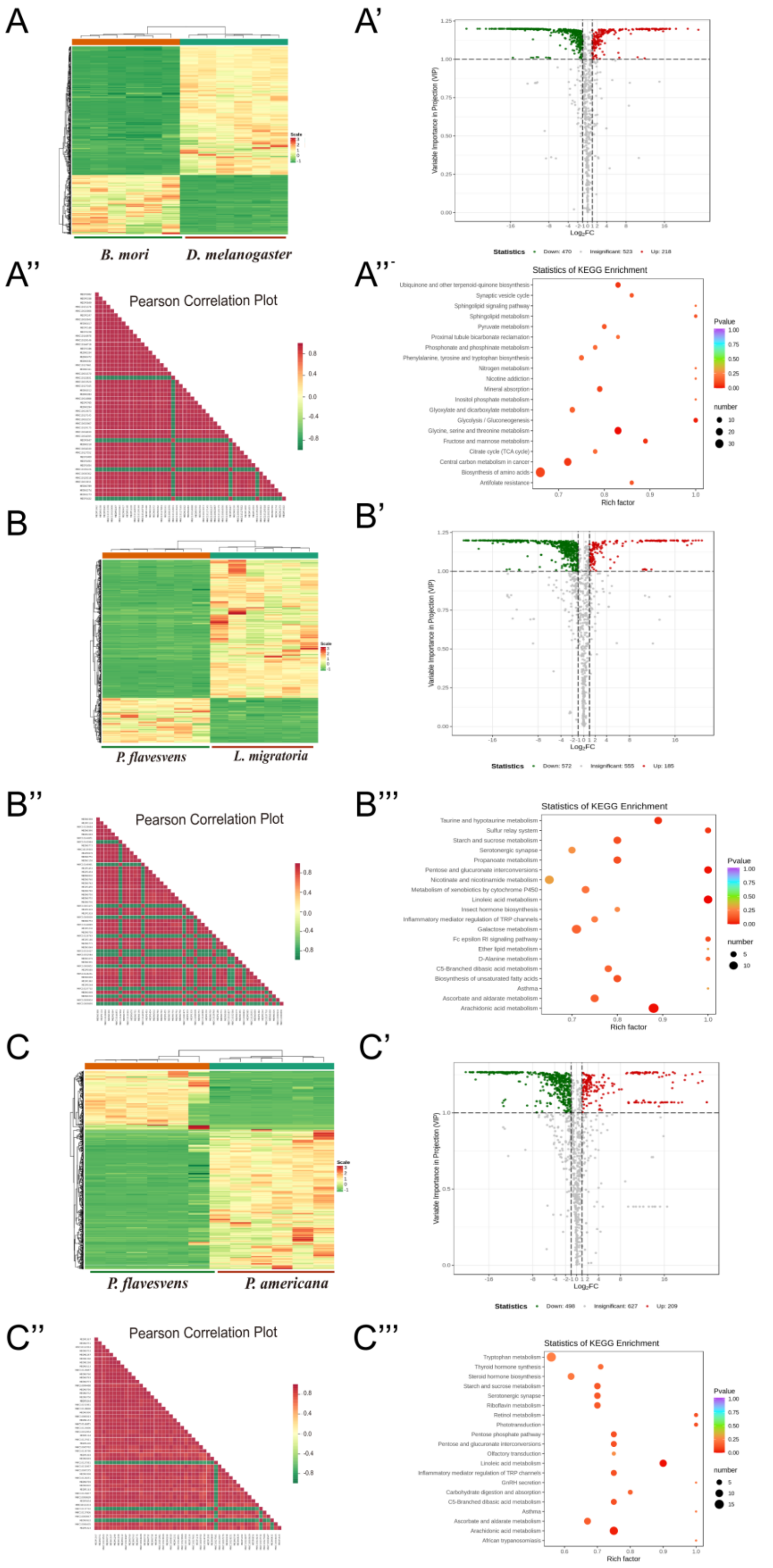

Figure S5. Heatmap, volcano plot, Pearson correlation plot, and KEGG enrichment of the metabolites between *B. mori* vs *D. melanogaster* **(A-A''')**, *P. flavesvens* vs *L. migratoria* **(B-B''')**, *P. flavesvens* vs *P. americana* **(C-C''')**.

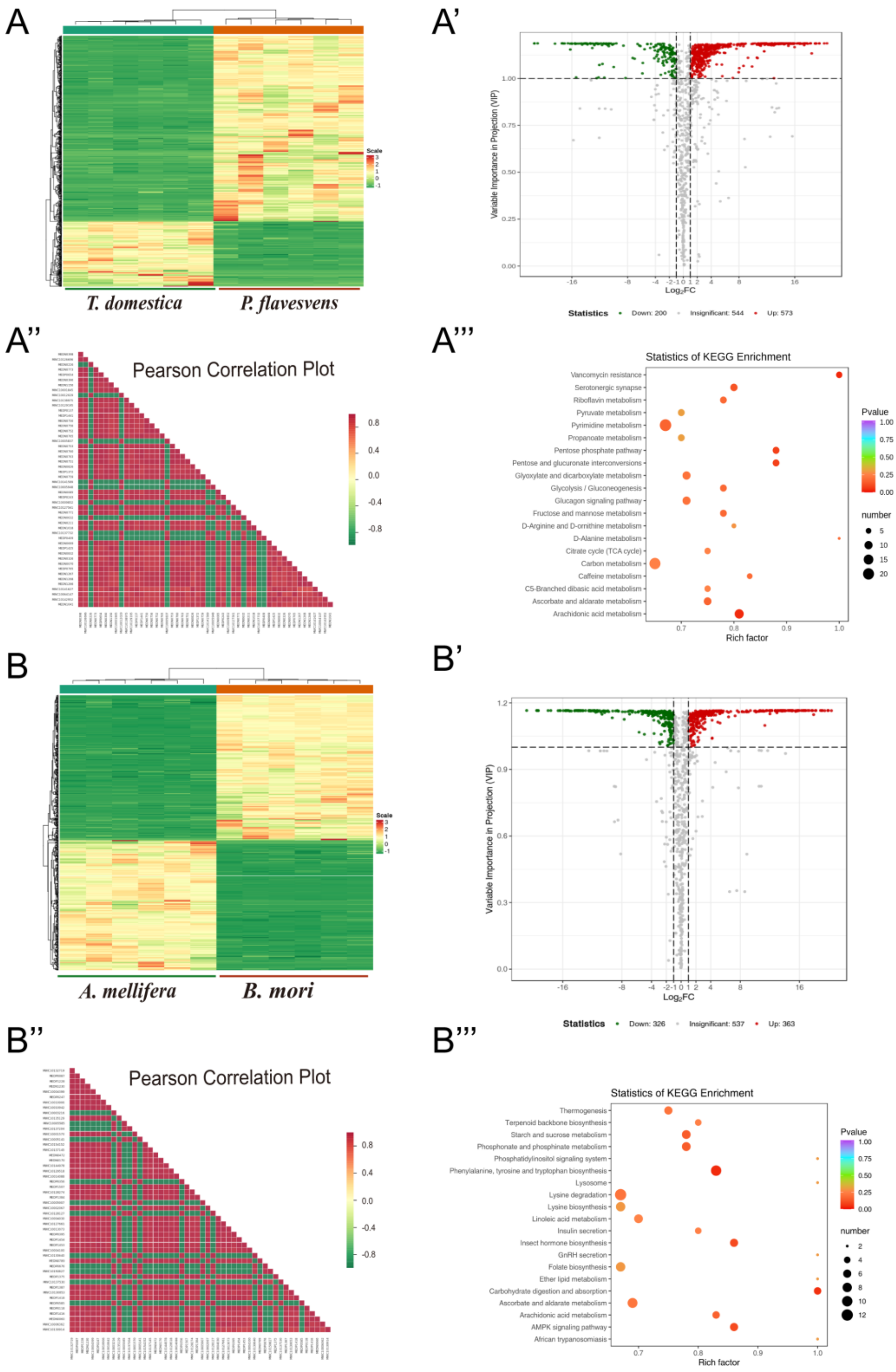

Figure S6. Heatmap, volcano plot, Pearson correlation plot, and KEGG enrichment of the metabolites between *T. domestica* vs *P. flavesvens* **(A-A''')**, *A. mellifera* vs *B. mori* **(B-B''')**.
